# Non-contrast enhanced visualization of the equine foot vasculature in a cadaver model using time-of-flight sequence

**DOI:** 10.3389/fvets.2025.1585940

**Published:** 2025-07-18

**Authors:** Bianca A. Underberg, Sabine Kaessmeyer, Daniela Schweizer, Barbara Drews, Elke Van der Vekens

**Affiliations:** ^1^Division of Clinical Radiology, Department of Clinical Veterinary Science, Vetsuisse Faculty, University of Bern, Bern, Switzerland; ^2^Graduate School for Cellular and Biomedical Sciences, University of Bern, Bern, Switzerland; ^3^Divison of Veterinary Anatomy, Department of Clinical Research and Veterinary Public Health, Vetsuisse Faculty, University of Bern, Bern, Switzerland

**Keywords:** MRI, angiography, TOF, horse, post-mortem perfusion, vascular anatomy, hoof

## Abstract

**Objective:**

The objective of this study was to establish a non-contrast enhanced MR angiography (NC-MRA) sequence for the equine foot (EF) using a post-mortem angiography model.

**Materials and methods:**

Time-of-flight (TOF) sequences were tested using variable parameter settings and 3 slice orientations during vascular perfusion of frozen–thawed cadaver EF with paraffine oil. Transverse and dorsal orientations were planned perpendicular or parallel to the sublamellar vascular plexus at the dorsal aspect of P3, or approximately perpendicular to the coronary plexus. Visibility of the coronary plexus, sublamellar plexus, palmar plexus, terminal arch and its respective branches, solar plexus, and the marginal circumflex vessels was evaluated in a total of 74 sequences.

**Results:**

Twelve EF of 10 horses were scanned. Visibility of vessels as small as the sublamellar plexus was best achieved by 3D-TOF sequences in transverse and dorsal orientations with the following parameters: pixel size 0.34 × 0.48 mm, slice thickness 0.2 mm, interslice gap 0.2 mm, TR/TE 21.2/4.7 ms, flip angle 16°, TONE ramp 70%, acquisition time 22.05 min. Only for the sublamellar plexus, the transverse acquisition showed a slightly better visualization than the dorsal acquisition, however the latter could include nearly the entire EF in the field of view with the same acquisition time.

**Conclusion and discussion:**

3D-TOF allowed visualization down to at least the sublamellar venous plexus which is reported to have diameters of approximately 0.45 mm. The post-mortem model facilitated establishment of a TOF sequence without the need for experimental animals.

## 1 Introduction

The vascularization of the equine foot (EF) is a complex system, whose importance is well-known, particularly regarding the pathophysiology of diseases like laminitis. Early diagnosis and treatment of acute laminitis are crucial, as these may allow restitutio ad integrum. Vascularization also plays a significant role in the healing of tendon, ligament and osseous lesions and the delivery of drugs to the EF (1–4). Therefore, early detection of vascular impairment can be life-saving, as it can lead to a better prognosis for the affected EF (5).

While magnetic resonance imaging (MRI) has become the modality of choice to investigate soft tissue pathologies in the EF, the standard sequences do not allow good visualization of the blood vessels and neither does native computed tomography (CT) (6, 7). Currently, equine orthopedic contrast-enhanced CT (CE-CT) and MRI (CE-MRI) examinations are performed focusing on soft tissue enhancement rather than visualization of blood vessels (8–12). Specific CT-angiographic (CTA) studies of the EF are limited and mainly serve as anatomical vascular reference, to evaluate perfusion after severe trauma and to assess necrosis (13, 14). In addition, restoration of flow was achieved in a post-mortem model that allowed the description of the vascular anatomy by CTA (15).

The use of non-selective, systemic, intravenous CE-MRI and CE-CT are compromised by the large equine body weight, requiring large volumes of contrast agents. This is associated with high costs, specifically for MRI where typically gadolinium is used (9, 16). This limitation can be overcome by intra-arterial regional perfusion, as this approach requires less contrast agent and provides higher contrast enhancement of soft tissue lesions and blood vessels, in the EF and in the equine brain (9, 11, 17). However, placing intra-arterial regional catheters within the high-field magnet is complicated and requires expensive MRI-compatible materials. This not only increases costs but also raises safety concerns regarding the procedure (18). Although rare, minor adverse effects associated with the administration of iodinated and non-iodinated contrast media have been reported in horses undergoing CT or MR examinations, respectively (10, 16, 19–21). The most commonly used contrast agent in MRI is gadolinium, which generally presents a lower risk of adverse effects compared to iodinated agents used for CT examinations. However, the injection of a contrast medium and associated risks and costs can be avoided by using non-contrast enhanced MR angiography (NC-MRA) (22).

NC-MRA methods either utilize flow such as time-of-flight (TOF) and phase-contrast (PC) MRA sequences to distinguish signals from moving blood and stationary tissues, or steady-state free precession MRA techniques, that visualize blood vessels because of the difference in T2/T1 ratio between blood and most other tissues (23, 24). TOF-MRA is well established in human medicine and can be used for the visualization of intracranial blood vessels with very small diameters down to 0.3 mm in 3 T systems and even 0.14 mm in 7 T, limited rather by pixel resolution than blood flow velocities (25, 26).

In equine veterinary medicine, the use of a TOF-MRA protocol was only described for visualization of the vasculature of the equine head. It showed a subjectively better spatial and contrast resolution than PC-MRA (27). To the authors knowledge, no TOF-MRA protocols for the EF have been published until now.

In the EF, arteries branching from the terminal arch are reported to have a diameter of 0.7 mm using conventional angiography (28). The diameter of the blood vessels of the sublamellar bed has been described as approximately 0.45 mm for veins and 0.015 mm for arteries using histology (29).

Recently, our group established a post-mortem model, allowing restoration of vascular flow in frozen–thawed equine distal cadaver limbs, without any special prior preparation. With this model, perfusion down to the sublamellar plexus was visualized using CE-CT and lipophilic contrast mixed in paraffine oil (15). As it mimics the in-vivo vascular flow, we hypothesized, that this model could allow ex-vivo perfusion studies and the development of MRA sequence, at the same time avoiding repetitive anesthesia of research horses, which is in line with the 3R principle.

Therefore, the purpose of this study was to prove that the post-mortem vascular cadaver model of the EF can be used to develop NC-MRA sequences. A second aim was to create a TOF-MRA sequence visualizing the blood vessels of the EF as small as the sublamellar vascular bed.

## 2 Materials and methods

Equine forelimbs were derived from horses euthanized at the ISME Equine Clinic of the Vetsuisse Faculty, University of Bern, Switzerland between 2021 and 2023 for clinical reasons unrelated to this study. Only limbs of horses without a history of lameness associated with the distal limb were collected and disarticulated at the level of the middle carpal joint. All owners signed an informed consent form permitting the use of tissues and images for research purposes.

### 2.1 Vascular perfusion model

#### 2.1.1 Freezing — thawing process

All limbs were prepared and positioned for MRA scanning based on a previously published protocol for a frozen–thawed EF vascular model (15). In brief, the limbs were frozen within 24 h after death for at least 2 weeks at −20°*C.* Prior to freezing the EF were cleaned, all inorganic and metallic material removed, and the coat was clipped. To exclude limbs with marked osseous changes, orthogonal radiographs of the foot and fetlock were performed and evaluated by a board-certified veterinary radiologist (EVdV). Before MR imaging, the limbs were thawed for approximately 12–24 h at room temperature.

#### 2.1.2 Catheterization

After thawing the limb, stainless steel, MR-compatible, 18 G cannulas [Veterinär-Kanülen, Unimed SA, Lausanne, Switzerland] were inserted into the median artery and radial vein at the level of the disarticulated surface (middle carpal joint). They were secured with two simple surgical sutures each.

#### 2.1.3 Positioning

The limb was placed in the MRI unit in supine position on two supports: a foam pad, containing an indentation for positioning of the proximal metacarpus, and a rolled-up disposable under pad supporting the hoof. A flat plastic container was placed under the proximal end of the limb to collect fluids draining from the cut surface during the perfusion. A protective sheet was placed under and over the limb to avoid contamination of the MRI system and artifacts from leakage of the lipophilic perfusate.

#### 2.1.4 Tube and pump system

**Figure 1:**
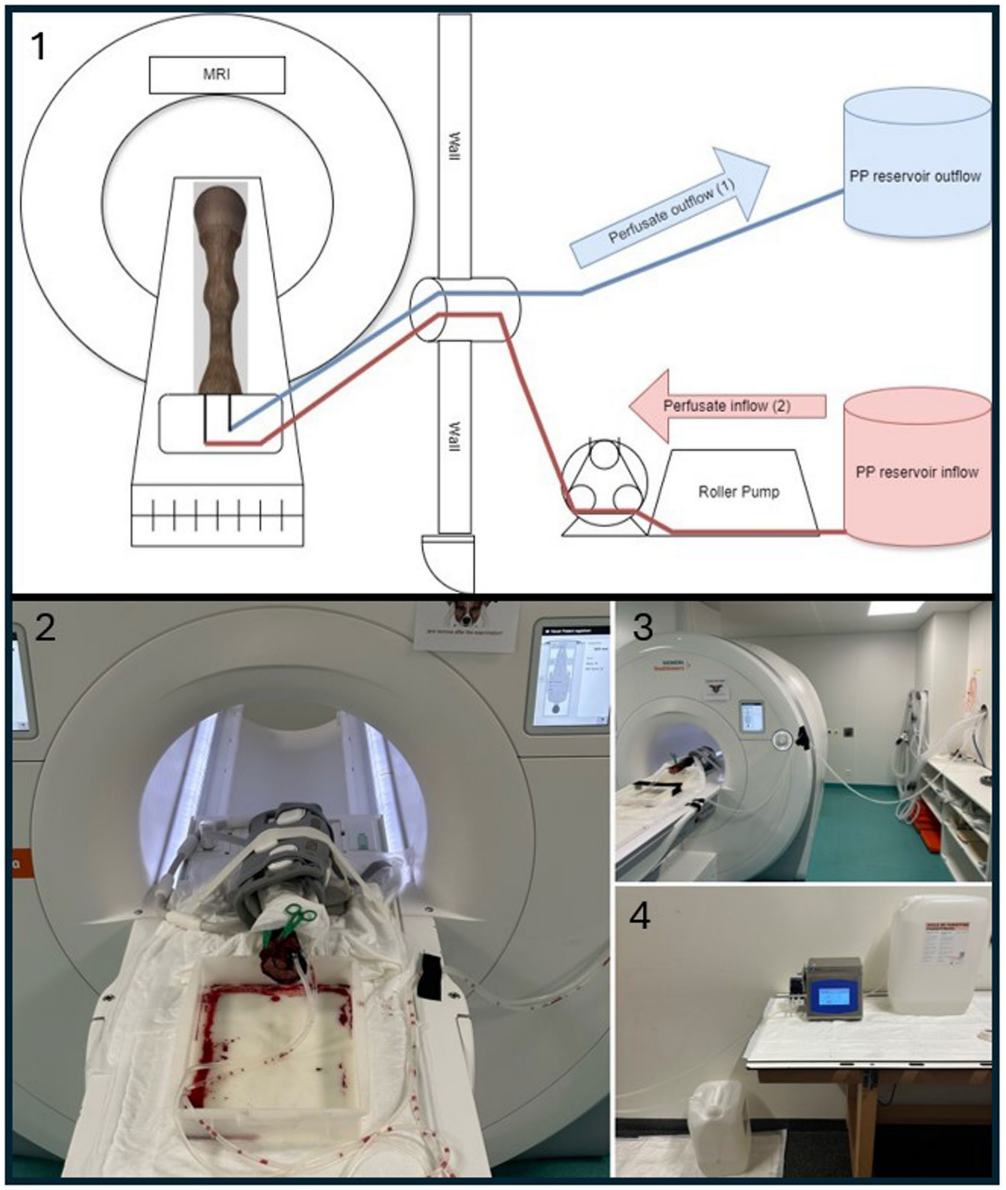
Experimental setup for ex vivo MRI perfusion imaging. 1 = Schematic drawing of the perfusion setup used in this study. The paraffinum perliquidum (PP) perfusate inflow (red) mimics arterial inflow (2) and is delivered via a roller pump. The perfusate outflow (blue) mimics venous outflow (1) and is collected in a separate reservoir. 2 = Corresponding photograph of the equine foot (EF) positioned in the MRI scanner. The EF is surrounded by the gray coil. A flat plastic container is visible under the proximal end of the limb containing some blood and perfusate draining from the cut surface during the perfusion. A larger draining vessel was closed by the green clamp. 3 = MRI setup showing the perfusion tubes running between the EF and the roller pump located outside the Faraday cage, passing through a tubular opening used for anesthesia tubes and lines in live animals. 4 = Perfusion setup with PP reservoir tanks and tubing connections, located outside the MRI room.

Two silicone tubes (each 3 mm inner diameter and 5 m length) [BiotoolSwiss AG, Kirchberg, Switzerland] were connected to both intravascular cannulas (A. mediana, V. radialis), to perfuse the vasculature as an open system. The arterial and/or venous tubes were connected to a roller pump [DosiPump DP 1000 BioToolSwiss AG, Kirchberg, Switzerland] placed outside the MRI room (Figure 1). Prior to their connection to the intravascular cannulas, the tubes were pre-filled with the lipophilic perfusate to avoid filling defects in the blood vessels caused by air-embolization. Immediately prior to the start of the first image acquisition, the limb was perfused via the arterial access for approximately 1 min, to flush out most of the remaining blood and blood clots. For all angiographic sequences, the perfusion with the roller pump was started together with the MRA scan acquisition. Therefore, the total perfusion time was approximately 1 min longer than the total image acquisition time. Perfusion was stopped during acquisition of non-angiographic sequences used for sequence planning and between individual angiographic sequences.

#### 2.1.5 Lipophilic perfusate

For the perfusion of the vasculature, a canister with a total volume of 20 L of paraffinum perliquidum oil [Huile de paraffin, Ideal Chimic SA, Carouge, Switzerland] was used as lipophilic perfusate. To fill the tubes, 517.9 mL of oil was required and for a total filling of the EF additionally approximately 92 mL was needed.

The perfusate was injected at a flow rate of 100 mL/min as proposed in a previous study (30). The viscosity of the paraffinum perliquidum oil was 24.864 mPa/s, to avoid leakage and/or embolization due to the post-mortem status (31, 32).

#### 2.1.6 Perfusion direction

The initial trials primarily focused on testing the perfusion setup and different perfusion directions. These included perfusion via the median artery in physiological direction (antegrade), perfusion via the radial vein in retrograde direction and arterial–venous combined perfusion via both vessels simultaneously.

### 2.2 MRA acquisition

For the MRA acquisitions, a 3 T unit (Magnetom Vida, Siemens Heathineers, Erlangen, Germany) was used.

#### 2.2.1 Coils

In all cases, either the 32- channel table-integrated human spine coil or an 18-channel surface coil were used as receiver coil. The body-coil was used as the transmit coil.

#### 2.2.2 Sequence type and acquisition parameters

Localizer images in three planes followed by an isotropic 3D-T1w gradient echo sequence (MPRAGE, TR 2300 ms, TE 2.98 ms, Flip angle 9°, voxel size 0.5 × 0.5 × 0.5 mm) were obtained from each limb to depict anatomical landmarks for more accurate planning. Variable NC-MRA sequences were then tested using one of the receiver coils. The standard manufacturer presets for 2D-TOF and 3D-TOF sequences from the human limb protocol served as the basis for the sequence development. The main parameters, in particular the time-to-echo (TE) and time-to-repetition (TR), flip angle (FA) and tilt-optimized non saturated excitation (TONE) ramp were kept within the recommended ranges published for 2D- and 3D-TOF sequences (33, 34). The influence of a saturation band with and without subtraction during post-processing was tested for potential differentiation between venous and arterial flow. Further adjustments aimed to achieve a higher resolution by increasing the number of blocks and slices per block. Also, the slice thickness was reduced in combination with a decrease in TR and the FA. Finally, to avoid exceeding specific absorption rate (SAR) limits, the parameters needed to be adjusted to a lower flip angle but a longer TR, along with the longer TE. The field of view (FOV) varied throughout the study based on the size of the EF and the slice orientation.

#### 2.2.3 Slice orientation

**Figure 2:**
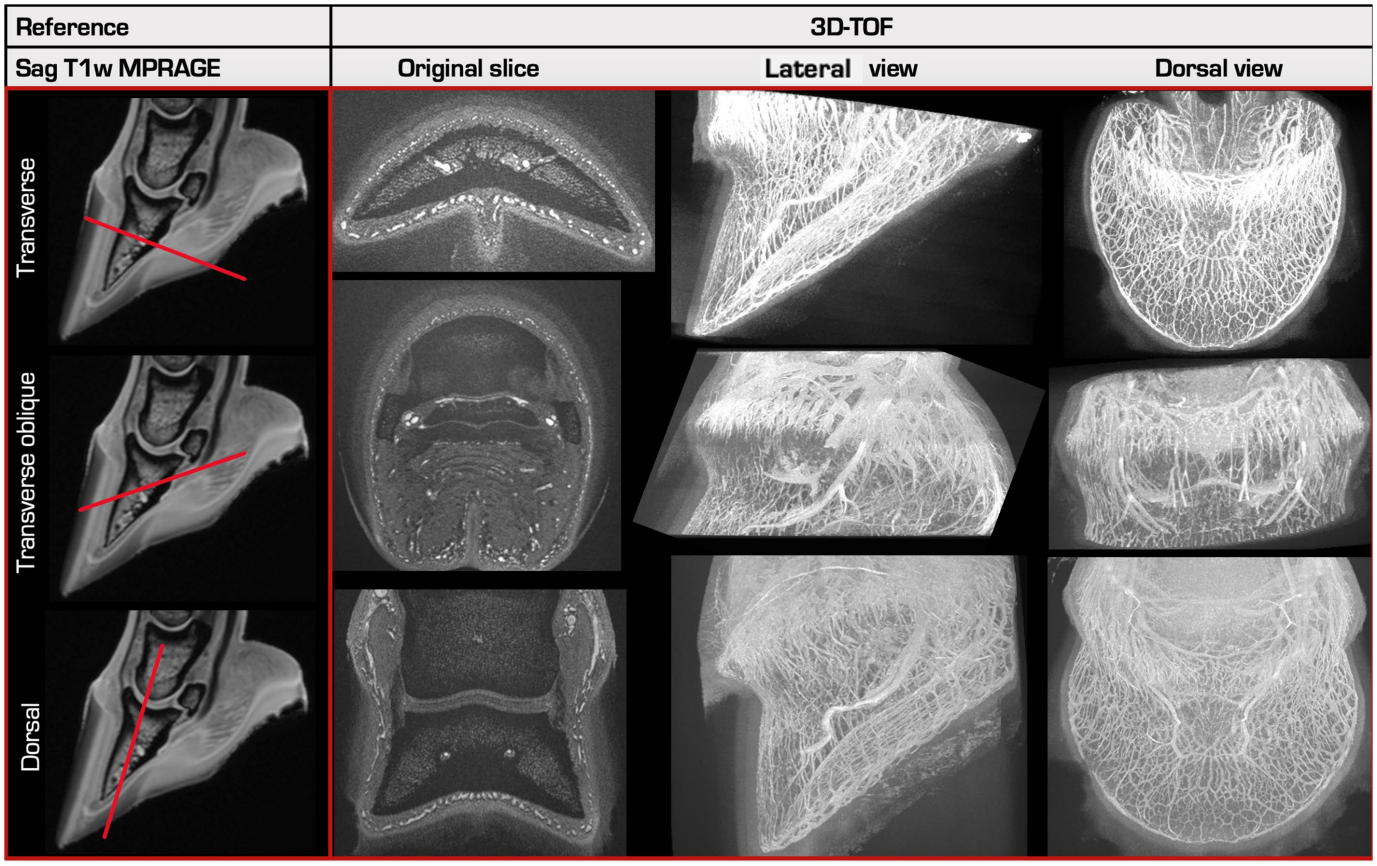
Overview of the 3 different scan orientations. A reference sagittal T1-weighted MPRAGE (Magnetization Prepared - Rapid Gradient Echo) image is shown in the left column, made prior to perfusion. 3D-TOF images created in 3 different slice orientations (right) with their respective 3D-Maximum Intensity Projections (MIP) in a lateral and in a dorsal view are shown in the other columns. The first and the last row show the normal transverse and dorsal slice directions, perpendicular or parallel to the sublamellar plexus at the dorsal aspect of P3, respectively. The middle row shows the transverse oblique slice direction, oriented approximately perpendicular to the coronary plexus at the dorsal aspect of P3.

All sequences were at least acquired in a normal transverse plane, perpendicular to the sublamellar plexus at a 90° angle to the dorsal border of P3 in the mid-sagittal view and included at least part of the sublamellar dermis. The best sequences were additionally acquired in two other slice orientations (Figure 2). They consisted of a transverse, slightly dorsodistal-palmaroproximal oblique plane (referred to as “oblique transverse” in the remainder of the text), approximately perpendicular to the coronary plexus; and a dorsal plane, parallel to the dorsal border of P3 in the mid-sagittal view.

#### 2.2.4 Image storage and review

The images were stored in DICOM format in the clinical PACS system (IMPAXEEServerRad, Agfa HealthCare, Mortsel, Belgium), and evaluated using a viewing software [IMPAX EE R20, Agfa HealthCare, Mortsel, Belgium].

### 2.3 MRA evaluation

For each MR sequence, the visibility of the vascular system was subjectively evaluated using the original sequences as well as multiplanar and maximum intensity projection (MIP) reconstructions of the raw data for the 3D sequences. The evaluation was performed in consensus by the first author (BU) and a board-certified veterinary radiologist (EVdV).

**Figure 3:**
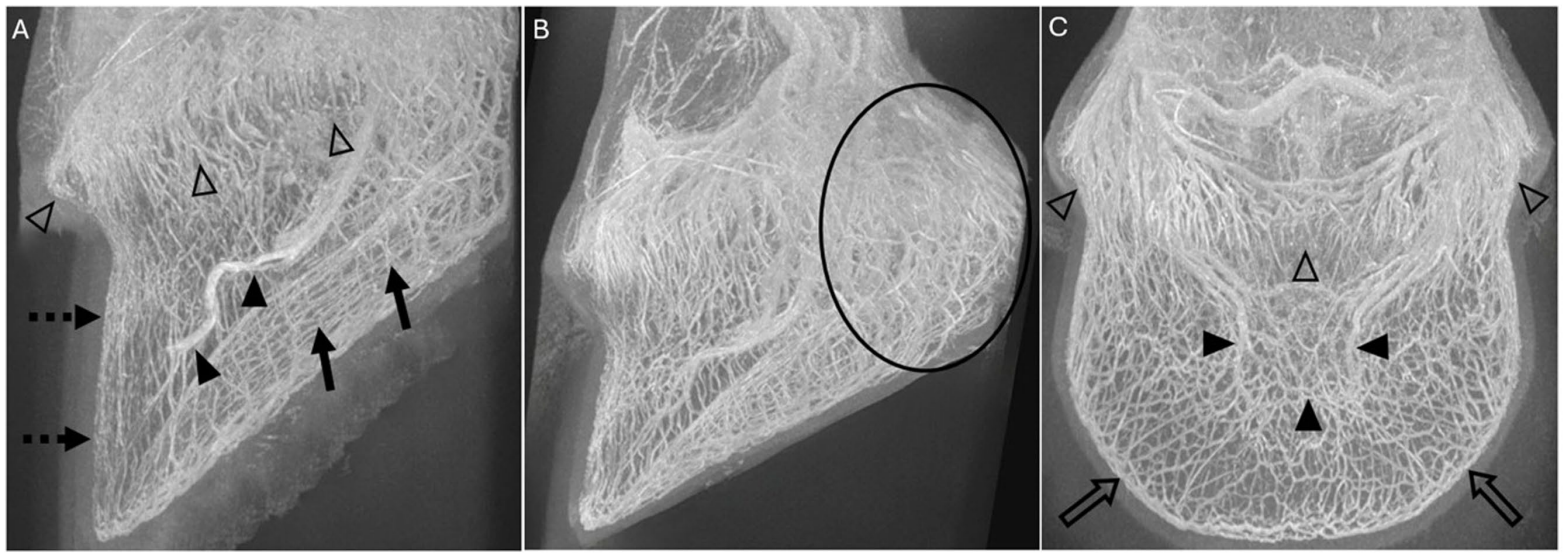
Volume rendered-MIP reconstructions of 3D-TOF sequences of limb 10 **(A)** and limb 12 **(B,C)** showing the 6 scored vascular plexuses: **(A)** The coronary plexus (open arrowheads), the sublamellar vascular plexus (dotted arrow), the solar plexus (solid arrows) and the terminal arch (solid arrowheads) are identified in this lateral view. **(B)** Lateral view, where the circle is surrounding most of the plexus of the palmar region (frog and ungular plexus). **(C)** The coronary plexus (open arrowheads), the terminal arch (solid arrowheads), and the marginal circumflex vessels (open arrows) are identified in this dorsal view.

The subjective visibility (good 2, intermediate 1, bad/absent 0) was assessed in the 6 regions containing a vascular plexus as described in previous studies: coronary plexus, sublamellar plexus, plexus of the palmar region (frog and ungular plexus), terminal arch and its respective branches, solar plexus, and the marginal circumflex vessels (16, 29, 35) (Figure 3).

#### 2.3.1 Sequence scoring and vascular visibility

The visibility of the blood vessels of the respective plexuses was evaluated and categorized based on their signal characteristics. Vessels with a continuous, well-defined, circular signal in transverse section, were defined as “good” and assigned a score of 2. Vessels that showed a focal interruption or narrowing of the diameter but otherwise presented a well-defined course, continuous over several slices, were classified as “intermediate” and assigned a score of 1. Finally, vessels with an interrupted course, ill-defined borders, or absent signal, were categorized as “bad” and scored with 0. For each sequence, the scores of the included plexuses were summated and ranked as percentages according to their result relative to the maximum score they could acquire. If all blood vessels were assigned a “good” visibility (score of 2) in all 6 regions, the maximum score was 12. If certain regions were not included in that scan sequence, they were marked as “X” and excluded from the calculation of the relative score.

Additional parameters recorded were extravasation, MRA artifacts, and the perfusion time.

### 2.4 Statistics

Variables were tested for normality using the Kolmogorov–Smirnov and Anderson-Darling tests. They were described by mean and standard deviation when normally distributed. The median and interquartile ranges were used in the event of skewed data. Categorial variables were expressed as counts and percentages. NCSS was used for data analysis (NCSS 2023 Statistical Software, LLC. Kaysville, Utah).

## 3 Results

This post-mortem study included 12 cadaver forelimbs of 10 different horses. Both forelimbs were used in 2 horses, only the left (5) or right (3) limb was used in 8 horses. The breed and sex distribution were five Swiss Warmblood, two Freiberger, one Quarter Horse, one Irish Sport Horse and one Friesian: four mares and six geldings. The mean age was 16.25 years with a range of 6 to 24 years. Catheterization and perfusion were successful in all cases.

### 3.1 Vascular perfusion model

A total volume of approximately 10 L of paraffinum perliquidum was required for a perfusion time of 90 min. All limbs were perfused with an antegrade perfusion direction. In limbs 1 and 2, a retrograde and in limb 2, the arterial–venous combined perfusion directions were performed in addition. During the retrograde perfusion and arterial–venous combined perfusion, image acquisition had to be stopped several times due to perfusion failures caused by tube ruptures and loosening of the tube connections. Therefore, retrograde perfusion and arterial–venous combined perfusion was abandoned after limb 2. The 12 limbs had a mean total perfusion time of 89.76 min with a range of 19.31 min to 209.1 min.

### 3.2 MRA acquisition and evaluation

#### 3.2.1 Coils

A total of 34 TOF sequences were performed using the 32-channel table-integrated spine coil and 40 sequences with the 18-channel surface coil. In sequences performed with identical parameters, except for the selected coil, the overall image quality was superior when using the 18-channel surface coil as the receiver coil compared to the 32-channel table-integrated spine coil due to its higher resolution.

The selection of the 32-channel table-integrated spine and or surface coils was automatically determined by the MR system. This had gone unnoticed in some scans. To prevent this automatic selection, the preset “ACS all except spine coil” was activated from limb 8 onwards.

#### 3.2.2 Sequence type and acquisition parameters

A total of 67 3D-TOF and 7 different 2D-TOF were performed. The ranges of scan time, voxel sizes and slice thickness are listed in Table 1. The parameters of the best performing sequences for each limb can be found in Table 2. The overall best performing sequences are listed in Table 3.

**Table 1:** Range of scan times, pixel sizes and slice thicknesses for both the 3D- and 2D-TOF sequences performed on 12 equine distal cadaver forelimbs using a 3 T MRI scanner.

Sequence	Scan time range (min)	Pixel size range (mm)	Slice thickness range (mm)
3D -TOF	5.5–23.08	0.34 × 0.48–0.70 × 0.74	0.1–0.5
2D -TOF	2.23–3.96	0.70 × 0.63–0.97 × 0.70	2.5

**Table 2:** The parameters of the best performing 3D-TOF sequence for each equine distal cadaver forelimb (Limbs 1–12).

Limb	Score %	Sequence	Slice orientation	Time of aqcuisition (min)	Slice thickness	Repitition Time (TR)	Echo Time (TE)	Flip angle°	Saturation Band	Tone rampe	3D Blocks	Slices per block	FOV (mm x mm)	Matrix	Voxel size (mm x mm)
1	50	3D TOF	TRA	7.18	0.5	27.3	3.42	19	proximal	70%	4	28	200 × 142.8	336 × 319.2	0.59 × 0.44
2	0	3D TOF	TRA	14.17	0.5	23.9	3.43	19	proximal	70%	9	28	172 × 122.8	336 × 319.2	0.59 × 0.44
3	16	3D TOF	TRA	12.6	0.5	23.9	3.42	19	proximal	70%	8	28	170 × 121.38	336 × 319.2	0.51 × 0.37
4	20	3D TOF	TRA	8.32	0.5	21	3.42	20	none	70%	6	40	200 × 181.20	384 × 364.8	0.52 × 0.50
5	41	3D TOF	TRA	6.03	0.5	20	3.11	20	none	70%	8	40	200 × 150	384 × 268.8	0.52 × 0.56
6	50	3D TOF	TRA	12.47	0.3	20.5	3.3	20	proximal	70%	8	64	200 × 143.8	384 × 268.8	0.52 × 0.53
7	70	3D TOF	TRA	22.05	0.2	21.2	4.7	20	proximal	70%	8	60	200 × 144.2	416 × 416	0.34 × 0.48
**8**	**90**	**3D TOF**	**TRA**	**22.05**	**0.2**	**21.2**	**4.7**	**16**	**proximal**	**70%**	**8**	**60**	**200 × 144.2**	**416 × 416**	**0.34 × 0.48**
9	75	3D TOF	TRA	22.05	0.2	21.2	4.7	20	distal	70%	8	60	200 × 144.2	416 × 416	0.34 × 0.48
**10**	**90**	**3D TOF**	**DORS**	**22.05**	**0.2**	**21.2**	**4.7**	**16**	**none**	**70%**	**8**	**60**	**200 × 144.2**	**416 × 416**	**0.34 × 0.48**
11	75	3D TOF	TRA	22.05	0.2	22.2	4.7	16	distal	70%	8	60	200 × 144.2	416 × 416	0.34 × 0.48
12	85	3D TOF	TRA	22.05	0.2	22.2	4.7	16	proximal	70%	8	60	200 × 144.2	416 × 416	0.34 × 0.48

The same sequence performed best in 2 limbs (Limbs 8, 10) as well as overall with a score of 90% of the possibly visible vascular plexuses well shown. They are highlighted in bold.

**Table 3:** The overall best performing parameters of all 3D-TOF sequences, scoring a minimum of 80% vascular visibility, performed on 12 equine distal cadaver forelimbs using a 3 T MRI scanner.

Limb	Score %	Perfusion time (min)	Slice orientation	Slice thick-ness	TR	TE	FA	Traveling saturation band	Tone rampe	3D Blöcke	Slices per block	FOV mm x mm	Matrix	Voxel size mm x mm
10	90	22.05	Dorsal	0.2	21.2	4.7	16	none	70%	8	60	200 × 144.2	416 × 416	0.34 × 0.48
8	90	22.05	Transverse	0.2	21.2	4.7	16	proximal	70%	8	60	200 × 144.2	416 × 416	0.34 × 0.48
12	85	22.05	Transverse	0.2	21.2	4.7	16	proximal	70%	8	60	200 × 144.2	416 × 416	0.34 × 0.48
8	80	22.05	Transverse	0.2	21.2	4.7	16	distal	70%	8	60	200 × 144.2	416 × 416	0.34 × 0.48
8	80	22.05	Dorsal	0.2	21.2	4.7	16	none	70%	8	60	200 × 144.2	416 × 416	0.34 × 0.48
10	80	22.05	Transverse	0.2	21.2	4.7	16	proximal	70%	8	60	200 × 144.2	416 × 416	0.34 × 0.48

FA = Flip angle; FOV = Field of view; TE = Time to echo; TR = Time to repetition.

In short, to enhance spatial resolution, the number of blocks and slices per block were increased, while the slice thickness was decreased. Simultaneously, the TR/TE, flip angle, slice orientation, and the presence of a saturation band were optimized to improve contrast resolution and overall image quality.

The best vascular visibility was achieved in two 3D-TOF sequences with the same parameters, in transverse and dorsal orientation, respectively and tested in two different limbs (limb 8 and 10) (Figure 2). Both resulted in an identical and overall highest score of 9/10 (90%). They had the following parameters: pixel size of 0.34 × 0.48 mm, slice thickness 0.2 mm, interslice gap of 0.2 mm, TE 4.7 ms, TR 21.2 ms, flip angle of 16°, TONE ramp of 70% and having an acquisition time of 22.05 min (Table 3).

2D-TOF sequences had an overall lower score compared to 3D-TOF. The best 2D-TOF achieved a score of 1/12 (8%). In both 2D- and 3D-TOF sequences, a smaller voxel size was associated with overall better vascular visibility.

The distal and proximal parietal branches from the terminal arch could only be visualized in 3D-TOF sequences, but not in any of the 2D-TOF. In 6 out of 12 limbs (limbs 7 to 12) the proximal, distal or both parietal branches could be visualized in at least 1 sequence. From the 35 3D-TOF sequences performed in these 6 limbs, 33 included at least part of the terminal arch in the FOV. One or both branches of the terminal arch could be appreciated in 20 of them. In 7 of these 20 sequences (limbs 7 and 9) it was only possible to identify the distal parietal branch, 12 of 20 sequences showed the proximal and distal branches (limbs 8, 9 and 10, 12). One sequence (limb 11) depicted only the proximal branches. All sequences with an overall score of at least 70% or higher, showed both the proximal and distal branches of the terminal arch, except the latter that depicted only the proximal branches despite an overall score of 75%. The sequences showing only the distal branches had lower overall scores (60–70%). All sequences where neither the proximal nor the distal branch could be visualized, had a score of less than 60%.

#### 3.2.3 Slice orientation

All but 11 sequences were oriented in the normal transverse orientation, perpendicular to the sublamellar bed. They performed best for visualization of this vascular plexus but showed a poorer visibility of the coronary plexus compared to both other orientations.

The dorsal and the oblique transverse planes resulted in a slightly better visibility of the coronary plexus compared to the normal transverse plane, with the former performing best for this region. However, they performed slightly less good for the sublamellar bed compared to the normal transverse plane.

When comparing the number of plexuses included in the FOV for sequences that differ only in slice orientation, the dorsal orientation could include all anatomical regions of the EF. For the same scan time, the normal transverse orientation could fully include the sublamellar and solar plexus, the marginal circumflex vessel, the terminal arch, and the dorsal aspect of the coronary plexus, but not the palmar plexus or the palmar aspect of the coronary plexus.

As the oblique transverse sequences were made with a smaller FOV to decrease the scan time, only the coronary plexus and the most proximal aspect of the sublamellar vascular bed were included.

Both best performing 3D-TOF sequences, described below, had equal parameters and differed only in slice orientation (transverse vs. dorsal).

#### 3.2.4 Saturation band

The effect of a traveling saturation band was tested by placing it proximally in 32 sequences and distally in 13 transverse 3D-TOF sequences. In two dorsal 3D-TOF sequences a dorsal (anterior) and palmar (posterior) saturation band was placed, attempting to visualize the venous or arterial systems, respectively. Twenty-seven sequences were tested without the use of any saturation band. Among the three highest scored sequences, the two transverse had a proximal saturation band while the third one, with a dorsal orientation, did not have a saturation band. When comparing sequences with the same parameters, transverse sequences with a proximal saturation band had a slightly better visibility of the vasculature. Visualization of only the venous or the arterial system in the EF could not be achieved in any of the TOF sequences.

#### 3.2.5 Perfusion time and extravasation

In the final limb, both best performing sequences were scanned three times to see the impact of perfusion time on image quality, considering the flush-out of blood clots and extravasation. Scans after a 40 min perfusion time resulted in the subjectively best image quality, but the differences between both scan orientations remained as described above. The best image quality in a transverse sequence occurred after approximately 40 min (scanned as 3^rd^ sequence), while the best dorsal sequences occurred after 20 min (scanned as 2nd sequence).

Extravasation was observed in four out of 12 limbs (limb 1, 6, 8 and 12) after a mean scan time of 68.57 min (SD ± 7.5 min). In limb 1, extravasation was first observed after 40.61 min of a total perfusion time of 57.63 min. In limbs 6, 8 and 12, extravasation was seen after 96.81, 66.25, and 70.6 min perfusion time of a total perfusion time of 104.15, 209.01, and 114.46 min, respectively.

#### 3.2.6 Artifacts

**Figure 4:**
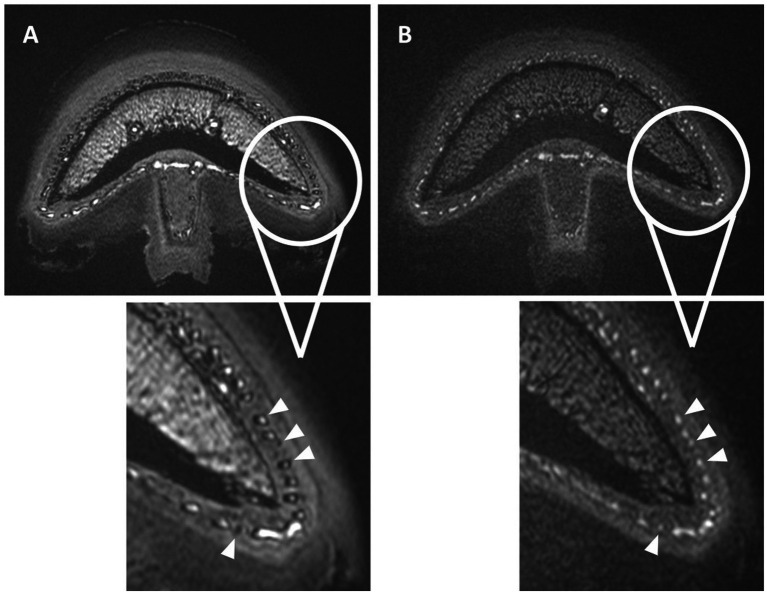
Normal transverse 3D-TOF images of limb 8 with **(A)** and without **(B)** the occurrence of the chemical shift type-two-artifact in all blood vessels (arrowheads). The distomedial aspects of the foot are enlarged in both scans, with a thick hypointense rim seen in the periphery of all vessels or completely obscuring the vascular lumen in image **(A)**. Adjusting the TE from 3.6 ms **(A)** to 4.8 ms **(B)** resolved this artifact as seen in image **(B)**.

Vascular visibility in all TOF sequences performed in the first 7 limbs was impaired by an artifact characterized by a thick, markedly hypointense rim (black ring) at the periphery of all blood vessel lumens. Specifically, the hyperintense lumen of the smallest vessels, such as the sublamellar bed, showed a marked decrease in diameter or was even completely obscured by this artifact. This was initially thought to be a susceptibility artifact caused by the post-mortem status of the limbs. However, this artifact represented a chemical shift type-two-artifact caused by phase cancelation of the signal and was subsequently resolved by changing the TE to be close to an “in-phase” time frame of fat and water in 3 T magnets (Figure 4).

In addition, in all sequences, a shine through artifact was visible in the 3D MIP reconstruction at the level of medullary cavity of P3, P2 and P1, when these regions were included.

## 4 Discussion

### 4.1 Vascular perfusion model

This study successfully proved the feasibility of developing a NC-MRA TOF sequence using a post-mortem cadaver model. It allowed the visualization of the EF vasculature as small as the sublamellar vascular bed and branches of the terminal arch.

This established model used frozen–thawed EF, without any prior preparation. It allows the restoration of vascular flow using paraffinum perliquidum to mimic in-vivo blood flow, enabling ex-vivo, but *in situ* perfusion studies. Thereby this equine cadaver model for EF angiography provides a cost and time-effective alternative or adjunct to in-vivo and tissue-based models. This, in line with the 3R principle, avoids repetitive anesthesia of research horses which is otherwise essential to develop such a protocol. In addition, it makes the planning of research more flexible. The use of lipophilic solutions for post-mortem angiography is well established in human forensic medicine (36, 37). Despite the ongoing autolysis after death, including loss of endothelial cell junctions and associated increased permeability of the vasculature, the lipophilic perfusate still remains intravascular and can flush out blood clots if the viscosity of the perfusate is chosen appropriately (36, 37). In our study, a paraffinum perliquidum perfusate with a viscosity of 24.86 mPas was used, previously described as the optimal value for post-mortem perfusion (31). The viscosity of blood in a healthy horse was previously reported with 7.1 +/− 2.3 mPas (38). Although our perfusate had a threefold higher viscosity compared to blood, previous studies showed that it could perfuse even the capillary beds of feline kidneys which are comparable in size to the capillary bed of the EF (29, 32).

The 3D-TOF sequence established in our study visualized the flowing perfusate up to the sublamellar vascular plexus using an antegrade perfusion direction. Retrograde perfusion and arterial–venous combined perfusion resulted in a perfusion failure and rupture of the tube system. This failure was likely due to excessive vascular pressure that developed secondary to the prolonged retrograde perfusion, associated with the presence of vascular valves in the proximal and distal aspects of the limb (2). In the CTA study from Blaettler et al., the arterial–venous combined perfusion direction resulted in the best image quality. However, this was the only flow direction scanned during perfusion (dynamic) of the model, as perfusion was stopped prior to scanning for the arterial and venous phases. Possibly scanning during perfusion and not the flow direction resulted in the better image quality of the arterial–venous scans in that study. In addition, their perfusion times were rather short when compared to our study, with a total of 10 min per limb and less than 2 min per phase, which likely explains the lack of system failure in their study (15).

### 4.2 Perfusion time and extravasation

We observed an improved image quality with increasing perfusion time, with the best visibility after about 40 min perfusion. Therefore, one can consider an improvement of vascular visibility at least up to a perfusion time of 40 min.

However, also the risk of extravasation (leakage) increases with increasing perfusion time, specifically above the mean of 68 min in this study. Therefore, further research may be needed to determine the optimal perfusion period.

### 4.3 Slice orientation

The normal transverse slice orientation, perpendicular to the main flow direction and vessel alignment of the sublamellar bed was tested first as this was considered the most challenging region for vessel visualization. This orientation was chosen as TOF-MRA utilizes repetitive radiofrequency pulses (RF pulses) that result in a relative saturation of the stationary tissue within the imaged slice, leading to low signal, while unsaturated protons flowing into the slice exhibit a high signal. This normal transverse orientation remained the best for the sublamellar vascular bed. Similarly, the oblique transverse plane performed slightly better for the coronary plexus. This in accordance with previous reports stating that keeping the imaging plane as perpendicular as possible to the direction of blood flow was a key factor of TOF sequences, to avoid saturation of flow parallel to the image plane (23, 39). Surprisingly, the 3D-TOF in a dorsal orientation performed nearly as good as the normal transverse for the sublamellar bed and the oblique transverse orientation for the coronary plexus and as good at the normal transverse for the other plexuses. As this plane is oriented rather parallel to the main flow direction in most plexuses, the good image quality of this orientation was unexpected. Possibly, the interconnections within the plexuses create a non-strict distoproximal flow pattern, allowing for partially oblique or even perpendicular flow through the plane, which could contribute to this result. Nevertheless, as discussed below, the TOF sequence is not as flow dependent as previously assumed but rather influenced by image resolution explaining our observation. In addition, previous studies suggested that with slow blood flow velocities, the inflowing fresh blood may become saturated before leaving the imaging slice. This would result in signal loss and was suggested to be the main limiting factor for imaging small vessels, but this was not observed in our study (34, 40, 41).

Although the sequence with the best parameters achieved the highest score of 90% in both the normal transverse and the dorsal orientation, the former had a subjectively slightly better visibility of the sublamellar vascular bed, while the latter showed better the vasculature of the coronary plexus. This is in accordance with the scan orientation being more perpendicular relative to the vascular flow within the coronary or sublamellar vascular plexus when orientating the slices in dorsal or transverse planes, respectively. However, in the dorsal orientation a larger part of the EF was included in the FOV, allowing visualization of all vascular plexuses of the EF without an increase of the scan time compared to the normal transverse orientation. Therefore, the authors prioritized the dorsal orientation as the preferential scan plane for the 3D-TOF sequence.

### 4.4 Sequence type and acquisition parameters

Both, 3D-TOF and 2D-TOF are well described for the brain in both human and veterinary medicine (42–46). Most studies indicated a preference of 3D-TOF over 2D-TOF sequences for vessels with a fast blood flow, such as multi-directional intracranial vessels, due to their higher spatial resolution and increased SNR. For imaging of slow flow in a single direction, specifically for imaging peripheral arteries, 2D-TOF sequences were commonly used in most human literature (22, 47). On the contrary, Panda et al. stated that 2D-TOF sequences were preferred for fast arterial flow (23). In horses, TOF sequences have only been reported for the head so far (27). In that study 2D-TOF, 3D-TOF and PC were tested using a 0.23 T system. Their 2D-TOF provided the best image resolution and background suppression, but no hypothesis was given to explain this observation. In our study using a 3 T magnet, 3D-TOF showed a better vascular visibility compared to 2D-TOF sequences. None of the 2D-TOF sequences showed the vasculature branching from the terminal arch. This limitation was likely attributed to the lack of spatial resolution due to larger voxel size for 2D-TOF as shown in previous studies (42, 47). This assumption is in accordance with a recent 7 T MRI study on TOF-angiography demonstrating that the main limiting factor for visualizing small blood vessels was rather the spatial resolution of the imaging modality itself than the slow blood flow (26). That study proved that 3D-TOF sequences could show vessels with diameters as small as 0.14 mm, if the voxel size is equal to or smaller than the diameter of the vessel (25).

As mentioned in the introduction, the diameters of sublamellar arteries are 0.015 mm, while they are 0.45 mm for sublamellar veins (29). Therefore, we assume that in our study only the venous vasculature of the sublamellar plexus is visualized. The even smaller lamellar vessels could not be visualized in our imaging setup as the vessels with the largest diameter at that level are the axial veins. Their diameter of 0.07 mm - 0.18 mm is well below our voxel size of 0.34 × 0.48 × 0.2 mm^3^ (29). However, this confirms that we were able to show vessels of approximately 0.4 mm using our 3D-TOF sequence, which is similar to the vessel size visualized in the human brain using *in vivo* TOF-MRA using a 3 T magnet (25). In our study, the main improvement in image quality of the 3D-TOF sequence could be achieved with the reduction of voxel size by consecutive decreasing slice thickness, increasing the matrix, increasing the number of blocks, and increasing the slices per block. Downside of this development was the increased acquisition time of the sequences to maintain the SNR, ending up with an acquisition time of 22.05 min for the best performing sequences. Although this is a long sequence, the authors still consider it feasible when the clinical signs could be the result of a perfusion problem. In addition, decreasing the FOV to a specific region of interest shortens the scan time of the sequence.

The SAR of the 3D-TOF sequences was high compared to 2D-TOF sequences but reducing the flip angle (20 to 16°) or increasing the TR resulted in lower SAR (as low as 0.3) and did not affect the image quality.

### 4.5 Saturation band

While the inability to display the sublamellar arteries in our TOF sequences could be explained by reaching the limits in spatial resolution of the 3 T system, no specific visualization of either the palmar digital arteries or veins based on the flow direction was successful in this study. This failure to differentiate between the venous and arterial flow with the use of a traveling saturation band, might be due to a failure in synchronization of the blood flow velocity with the traveling saturation band and slice read out. Specifically, due to the post-mortem nature of this study, the use of cardiac gating was not possible. Further possible pitfalls were the lipophilic perfusate, the multidirectional in-plane course of the vessels, long acquisition time with multiple consecutive acquisitions, the smaller size of the arterial vascular system and the limited image resolution (24). These factors contributed to a lack of saturation of the perfusate within undesired arteries or veins, respectively. This was confirmed during subtraction of sequences with distal or proximal traveling saturation band from sequences without a saturation band, which did not result in a differentiation of venous or arterial blood vessels.

### 4.6 Artifacts

A major challenge during the study was to identify the cause of the black rings, which were mainly located within the vascular lumen and obscured the hyperintense signal of the perfusate, as chemical shift type-two-artifact. These artefacts, also known as India ink or black boundary artifact, occur in voxels with roughly equal amounts of fat and water. When the fat and water spins within these voxels are 180° out of phase at certain TEs, it causes the cancelation of signal. Once the nature of this artefact was identified, it was easily overcome by adjusting the TE, so the spins were in-phase for our 3 T system (4.7 ms), which was slightly different from the previously reported (2.4; 4.6; 6.8 ms) (46, 48, 49). The possible occurrence as well as the prevention of this artifact should be considered when using cadaver models in combination with lipophilic perfusates for future MRA studies. The shine through artifact of the medullary cavity was believed to be enhanced by the post-mortem condition, resulting in high protein byproducts giving an increased T1 signal and is considered an inherent limitation of the model (49).

## 5 Limitations

Although this post-mortem model offered clear benefits over the use of living experimental animals for sequence development, our proposed 3D-TOF sequence may need further optimization in live horses. Specifically possible benefits of a traveling saturation band to separate arterial and venous flow should be tested *in vivo*.

A final limitation of this study is the lack of immunohistochemistry of the EF blood vessels to confirm they were normal and support the MRA results. However, this was outside the scope of this study but should be evaluated in a future project.

## 6 Conclusion

In conclusion, frozen–thawed equine distal forelimbs without prior preparation and perfused with a lipophilic perfusate (paraffinum perliquidum) can be successfully used to develop NC-MRA sequences and for the visualization of EF blood vessels. The 3D-TOF protocol created in this study allowed for a high-quality 3D-visualization and detailed evaluation of the vascular architecture within the EF down to the sublamellar vascular bed. The NC-MRA method developed in this study on horse cadaver limbs not only allows blood vessel research on intact limbs but could potentially serve as an MRI model for pathologically altered EF, thus reducing the need for experimental animals in accordance with the 3R principle.

## Data Availability

The original contributions presented in the study are included in the article/supplementary material, further inquiries can be directed to the corresponding author.
